# Tobacco smoking among doctors in mainland China: a study from Shandong province and review of the literature

**DOI:** 10.1186/1617-9625-10-14

**Published:** 2012-09-24

**Authors:** Derek R Smith, Isabella Zhao, Lina Wang

**Affiliations:** 1 School of Health Sciences, Faculty of Health, University of Newcastle, Ourimbah, New South Wales, Australia; 2 Institute of Health and Biomedical Innovation, Queensland University of Technology, Brisbane, Queensland, Australia; 3 Department of Medicine, Longkou Chinese Medicine Hospital, Longkou, Shandong, China

**Keywords:** China, Chinese, Doctor, Physician, Smoking, Tobacco, Medical

## Abstract

**Background:**

Tobacco control represents a key area in which doctors can make a significant positive impact on their patients’ lives. Despite this fact, however, doctors in certain regions of China are known to smoke tobacco at rates similar to or even exceeding those seen within the general population.

**Objective:**

This study sought to investigate the smoking habits of doctors at a teaching hospital in Shandong province, as well as providing a brief review of smoking research that has been conducted among doctors elsewhere in China.

**Method:**

An anonymous questionnaire survey was distributed to doctors working at a university teaching hospital in 2008, as part of a larger study of occupational health issues in the healthcare profession.

**Results:**

The overall smoking prevalence rate of doctors in this study was 36.3% with significant differences observed between the genders (males: 46.7% and females: 5.3%). Age and total career length were also correlated with smoking habit, although no significant associations were found with department of employment.

**Conclusions:**

Overall, our study suggests that smoking rates among doctors in Shandong province are higher than those documented in many other countries, a finding which is consistent with previous research conducted in some other Chinese provinces. Addressing this issue from an intrinsic cultural perspective will clearly need to form the cornerstone of tobacco control efforts within the Chinese medical community in future years.

## Background

There are around one billion smokers in the world today and up to half of them will eventually die because of their habit. Tobacco use kills over 5 million people per year and is the single most important cause of preventable death [1]. Doctors have a major role to play in addressing this threat by providing primary care, quit smoking advice, tobacco related education and so on [2]. As a result, tobacco control represents a key area in which doctors can make a significant positive impact on their patients’ lives. Despite this fact, however, doctors in certain countries are known to smoke tobacco at rates similar to or even exceeding those seen within the general population [3]. Smoking by doctors themselves represents a critical issue in role modelling, as patients may be inclined to ask ‘how bad could smoking be…if so many doctors smoke?’ [4].

China remains one of the highest per capita users of tobacco in the world. According to the WHO *Global Adult Tobacco Survey* (GATS) of 2010, around 53% of Chinese men and 2% of Chinese women currently smoke [5]. Research conducted among the Chinese medical profession suggests that a large proportion of doctors are smokers, [6] while their smoking prevalence rate is only about 1/3 lower than that of the general population [7]. Despite these findings, little data exists on the smoking habits of doctors in Shandong province [8]. As such, the current study sought to investigate the smoking habits of doctors at a teaching hospital in Shandong province, as well as providing an overview of smoking research that has already been conducted among doctors in China and published in the international literature.

## Methods

As part of a larger study of occupational health issues, a cross-section of 200 healthcare professionals were surveyed at a university teaching hospital in Shandong province, China. The study was approved by the ethical review board of Longkou Chinese Medicine Hospital in Shandong Province, China and there were no penalties or rewards for compliance or non-compliance. Informed consent was implied if questionnaires were voluntarily completed and returned. Questionnaires were distributed during 2008 and collected within a 2-week period. All anonymous data were then entered into a spreadsheet program and analysed by statistical software. Basic prevalence rates were calculated, along with Pearson’s chi-square tests to ascertain statistical associations with smoking status. A review of published studies describing the smoking rates of Chinese doctors was also undertaken in 2012 using the National Library of Medicine’s (NLM) *PubMed* website [9]. Only English-language manuscripts were included. The reference lists of these manuscripts were then examined to locate any additional studies which had not been detected in the initial search.

## Results

**Figure 1:**
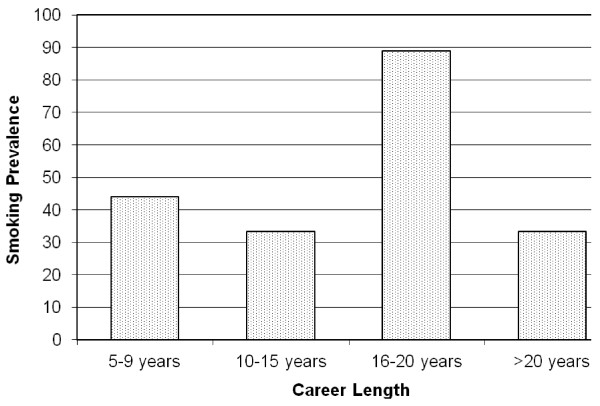
Smoking prevalence rates and career length.

An overall response rate of 93% was obtained from the multidisciplinary cohort of healthcare professionals surveyed. Of this group, 84 were medical doctors, among whom 79 (94.0%) provided information regarding their smoking habits. Almost three-quarters of the group were male (77.4%, n=65), with the majority (43.4%, n=36) aged 30 to 35Â years. Almost half (46.3%, n=37) reported working between 46 and 50 hours per week. Over one-third of the respondents (29 doctors, 36.3%) reported that they were current smokers, with 50 doctors (62.5%) stating that they had never smoked. Only one respondent reported having successfully quit smoking. There were statistically significant differences in smoking rates between the genders, with 46.7% of males being current smokers, compared to only 5.3% of females (P=0.0011). Age was significantly correlated with smoking habit (P = 0.0252) and no doctors under the age of 25 reported themselves to be a current smoker. Refer to Table 1. Total career length was correlated with smoking habit (P=0.0026) and no doctors who had worked for less than 5Â years were current smokers. Refer to Figure 1. Smoking was not correlated with department of employment (P=0.1987) although one of the highest rates was seen in orthopaedics, where exactly half the respondents were current smokers. Our literature review located a total of 16 studies of Chinese doctors that had been undertaken in various provinces between 1987 and 2011 and subsequently published in English. Refer to Table 2.

**Table 1:** Demographic correlations with smoking

	**Smoking Status**		
	**Never**	**Current**	**P for Trend**
**Gender**			
Male	53.3%	46.7%	-
Female	94.7%	5.3%	0.0011
**Age**			
25-29Â years	93.3%	6.7%	-
30-35Â years	54.3%	45.7%	-
36-40Â years	50.0%	50.0%	-
>40Â years	50.0%	50.0%	0.0252

**Table 2:** Smoking research conducted among Chinese doctors (published in English)

		** Current Smoker**^**a**^					** Study Details**		
**Location**	**Year**^**b**^	**All**	**Male**	**Female**	**Ex-Smoker**	**Never Smoked**	**Sample Size**	**Response Rate**^**c**^	**Authors**
Hubei	1987	30%	51%	5%	13%	57%	480	86%	Li & Rosenblood, 1996 [10]
Hubei	1996	45%	61%	12%	-	-	493	82%	Li *et al*., 1999 [11]
Hong Kong	2002	4%	-	-	2%	94%	757	19%	Abdullah *et al*., 2006 [12]
Hunan	2003	36%	-	-	11%	54%	658	80%	Yan *et al*., 2008 [13]
Various ^d^	2004	-	45%	2%	-	-	823	-	Yao *et al*., 2009 [14]
Hebei	2004	16%	32%	0%	1%	83%	286	79%	Smith *et al*., 2006 [15]
Various ^d^	2004	23%	41%	1%	3%	74%	3552	94%	Jiang *et al*., 2007 [16]
Hubei	2005	44%	58%	19%	-	-	347	87%	Li *et al*., 2008 [17]
Huhhot ^e^	2006	-	44%	0%	-	-	103	89%	Ceraso *et al*., 2009 [18]
Beijing	2006	-	55%	0%	-	-	103	89%	Ceraso *et al*., 2009 [18]
Guangzhou	2006	26%	46%	2%	-	-	945	61%	Lam *et al*., 2011 [19]
Guangxi	2007	26%	35%	3%	5%	69%	673	85%	Zhou *et al*., 2010 [20]
Fujian	2008	-	-	2%	-	-	685	76%	Wu *et al*., 2011 [21]
Shandong	2008	36%	47%	5%	1%	63%	200	93%	Smith *et al*., 2012 ^f^
Various ^g^	2009	10%	18%	4%	11%	79%	482	60%	Shi *et al*., 2010 [22]
Beijing	2010	29%	-	-	-	-	17	-	Shin *et al*., 2012 [23]
Various ^h^	2010-11	3%	-	-	2%	-	84	-	Zhang *et al*., 2012 [24]

^a^ Smoking rates rounded to the nearest whole number, ^b^ Year the study was conducted (not the publication year), ^c^ Response rates rounded to the nearest whole number, ^d^ Guangzhou, Chengdu, Wuhan, Tainjin, Harbin and Lanzhou (cities) located in Gansu, Guangdong, Heilongjiang, Hubei, Sichuan and Tianjin (provinces, respectively), ^e^ Inner Mongolia, ^f^ The current study, ^g^ National sample - Members of the Chinese Association of Anesthesiologists, ^h^ 84 doctors from 60 hospitals representing 20 provinces who were attending a tobacco control training course in Beijing.

## Discussion

Over one-third of doctors in the current study reported that they were smokers, with almost half of all males using tobacco. This relatively high smoking prevalence among doctors in Shandong is consistent with some previous research conducted in other parts of China and subsequently published in English, as shown in TableÂ 2. A study of doctors in Hunan during 2003, for example, documented exactly the same smoking rate as ours (36%) [13]. Higher smoking rates have been reported in Hubei, with figures of 44% to 45% overall, and 58% to 61% among males [11,17]. Similarly high smoking rates have also been documented among doctors in some other countries including Italy, Japan, Kuwait and the United Arab Emirates [3]. Lower rates of doctors’ smoking tend to be seen in countries with a more lengthy history of anti-tobacco activity [25].

The current study revealed significant differences in smoking rates by gender, which is consistent with virtually all previous smoking research conducted among doctors in China. Indeed, some investigations of Chinese doctors [15,18] and Chinese medical students [26] have reported having no females smokers at all. Many other investigations have documented smoking prevalence rates of less than 5% among female doctors and female medical students in this country [27]. Wide differences in prevalence rates are known to occur. One of the earliest studies, for example, appears to have been conducted in 1984 and documented a smoking rate of 57% among male doctors, but only 2% among their female counterparts [28]. Similarly, a study of Chinese cardiovascular physicians in 2008 found that females were far less likely to smoke when compared to their male counterparts (30% vs. <1%) [29]. Comparatively higher smoking rates have also been documented among Chinese medical students [30] and nurses [31]. This phenomenon may reflect a cultural reluctance for professional women to smoke in certain parts of the world, such as China. On the other hand, it may occur because women and children are generally exempt from many of the social situations where cigarette sharing is common in China [32].

Age was significantly correlated with smoking habit in the current study and no doctors under the age of 25 reported themselves to be a current smoker. Similarly, total career length was also correlated with smoking habit, with no doctors who had worked for less than 5Â years being current smokers. The first result is consistent with a previous study of Chinese doctors [15] where respondents less than 25Â years of age had the lowest prevalence. On the other hand, a previous study of Japanese doctors [33] found the highest smoking rate to be among doctors younger than 40Â years. In the current study, smoking was not correlated with department of employment although one of the highest rates was seen in orthopaedics, where exactly half the respondents were current smokers. Exactly how much a doctor’s medical specialty influences their smoking habits is uncertain, as previous research which examined this issue has provided inconsistent results [3].

Regardless of which department they may have worked in, when viewed from an international perspective, the overall rate of smoking among Chinese doctors in the current study was relatively high. This is in marked contrast to some other research where the lowest overall doctor smoking rates have been consistently documented in countries such as the United States, the United Kingdom, Australia and New Zealand [3]. Longitudinal investigations also suggest that smoking among doctors in these countries has been steadily decreasing over the past 30Â years [34-38]. Smoking rates among Australian doctors, while comparatively low, appear to have stabilised somewhat [39]. Tobacco use has been similarly declining among doctors in some other Asian regions. A recent examination of Japanese research, for example, elucidated a continuous decline of smoking among Japanese doctors since the mid 1960s [40]. Not all regions have demonstrated consistent, if any, declines in tobacco usage among doctors. For example, high rates of smoking have been documented in areas such as Greece, Italy and France; [3] suggesting that there is still much work to be done for tobacco control within the medical profession. Part of this effort would clearly need to encourage more doctors to quit their habit, as only one respondent in the current study reported that they had successfully quit smoking.

This finding is consistent with existing knowledge that quitting rates are known to be low within this group [6] – an issue which appears to encompass Chinese society in general. According to the WHO, for example, in 2010 only 16% of Chinese smokers planned to or were thinking about quitting smoking in the following 12Â months [5]. An earlier national smoking prevalence survey in China reported that while medical workers and teachers had relatively higher rates of cessation when compared to the general population, about half still did not intend to give up smoking [41]. Indeed, quitting is not the norm for contemporary Chinese smokers [42]. A study of Chinese health care providers in 2009 reported that around half lacked knowledge about nicotine replacement therapy [43]. This may suggest a lower awareness of tobacco control measures, generally, given that a recent study from Beijing [23] reported low levels of knowledge among doctors regarding the effects of smoking on Tuberculosis (TB), while many did not view smoking cessation as an integral part of TB treatment for their patients. Similarly, smoking among Chinese doctors is not necessarily a secretive behaviour, with one study reporting that almost 90% of health care providers who smoked did so in the wards, [13] while another study reported that 43% of Chinese surgeons had smoked in front of their patients [14].

It has been suggested that smoking is an imbedded part of Chinese medical culture and broader Chinese society [44]. Offering a cigarette ( or Fayan), for example, appears to have become a basic and highly ritualised feature within the Chinese medical profession, especially among male doctors [45]. This may contribute to smoking initiation and failure to quit [46]. Similarly, there is the issue of cigarette gifting from patients to doctors, [47] and the fact that for many years, smoking and the exchange of cigarettes has been associated with harmonious social interactions [48]. Chinese doctors may not have been setting a good example in this regard. One study from Changsha, for example, found that 68% of doctors would accept cigarettes offered by patients or their families, while 17% would smoke them [13]. Addressing these issues from an intrinsic cultural perspective will clearly need to form the cornerstone of tobacco control efforts within the Chinese medical community in future years.

While it has been suggested that China’s medical community may not have been active enough regarding tobacco control, [44] some positive moves have been occurring. The first Chinese stop-smoking clinic was established in Beijing in 1996 [24] for example, and in 2009 the Chinese health ministry launched a campaign to stop smoking among doctors and other medical workers [49]. These programs are clearly a step in the right direction and ones that need to be developed at all levels of the medical community. Educational institutions, such as teaching hospitals, occupy an ideal position to set positive examples in tobacco control [50].

As with all research projects, our study incorporated various strengths and limitations which are worthy of some brief discussion. Firstly, the data has certainly helped elucidate the smoking habits of doctors in Shandong province, China – a demographic and geographical subset from which limited data appears to have been published in English. Secondly, the literature review component provides an up-to-date summary of smoking research previously conducted in the Chinese medical community and published in English. One of the main limitations of our study might be the relatively small sample size and the fact that the data was sourced from only one hospital. Nonetheless, the hospital surveyed was one of the largest hospitals in the region, and one that services a mixed cohort patients from both metropolitan and regional areas. Furthermore, the study obtained a very high (>90%) response rate. As such, we are confident that these results provide a very useful snapshot of what tobacco usage habits might be among doctors in Shandong Province, China.

## Conclusions

Overall, the results from this study suggests that smoking rates among doctors in Shandong province are somewhat higher than those documented in many other countries, a finding which is consistent with previous research conducted in some other Chinese provinces.

## Competing interests

The authors declare that they have no competing interests.

## Authors’ contributions

DRS conceived the idea of the study and wrote the manuscript. IZ and LW collected the data and assisted in writing the manuscript. All authors read and approved the final manuscript.
